# Radiotherapy‐Induced Pleural Mesothelioma in a Childhood Leukaemia Survivor: A Case Report

**DOI:** 10.1002/rcr2.70342

**Published:** 2025-09-18

**Authors:** Sho Saeki, Rin Kurosawa, Akihiro Yamada, Minori Shimamura, Megumi Inaba, Takahiro Tashiro, Akiko Sakagami, Naomi Hirata, Yasumiko Jodai, Shinji Iyama, Yusuke Tomita, Takuro Sakagami

**Affiliations:** ^1^ Department of Respiratory Medicine Kumamoto Chuo Hospital Kumamoto Japan; ^2^ Department of Respiratory Medicine Kumamoto University Hospital Kumamoto Japan

**Keywords:** case report, leukaemia survivor, pleural mesothelioma, secondary malignancy

## Abstract

We report the case of a 41‐year‐old woman who developed an epithelioid‐type pleural mesothelioma (PM) decades after treatment for childhood acute myeloid leukaemia (AML), who was treated with chemotherapy and total body irradiation (TBI). The diagnosis was confirmed by thoracoscopic pleural biopsy and immunohistochemical staining. Although PM is classically associated with asbestos exposure, the patient had no known history of exposure. This case report highlights the fact that PM can occur as a late‐onset secondary malignancy following radiation therapy in childhood cancer survivors. Although radiation‐induced PM has been reported primarily in survivors of Hodgkin lymphoma or breast cancer, incidences following treatment for leukaemia are exceptionally rare. This case report highlights the importance of considering prior therapeutic irradiation, including total‐body irradiation, as a potential etiological factor for non‐asbestos‐related PM. It also emphasises the need for the long‐term surveillance and monitoring of childhood cancer survivors, particularly those who have received radiation therapy.

## Graphical abstract

Here, we report the development of pleural mesothelioma (PM) in a 41‐year‐old long‐term leukaemia survivor with a history of childhood leukaemia and total body irradiation (TBI). This observation highlights the importance of considering prior radiation therapy, including TBI, as a contributing factor in the development of secondary PM, especially in younger patients without a history of asbestos exposure.

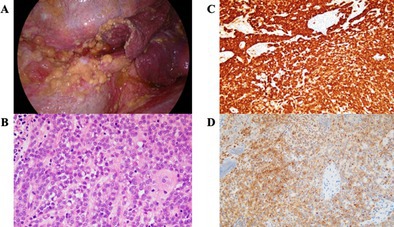

## 1 Introduction

Pleural mesothelioma (PM) is a rare but aggressive neoplasm primarily associated with asbestos exposure. However, PM can also develop in individuals with no known history of asbestos exposure, particularly those with a history of radiation therapy. This association has been most notably documented in survivors of Hodgkin lymphoma who underwent chest radiotherapy, with studies reporting an increased risk of PM several decades after treatment [1]. In contrast, PM following childhood leukaemia is extremely rare, with only one previous case reported in the literature [2]. Herein, we present the case of a 41‐year‐old woman who developed PM several decades after receiving chemotherapy and radiation therapy for childhood leukaemia.

## 2 Case Report

**FIGURE 1:**
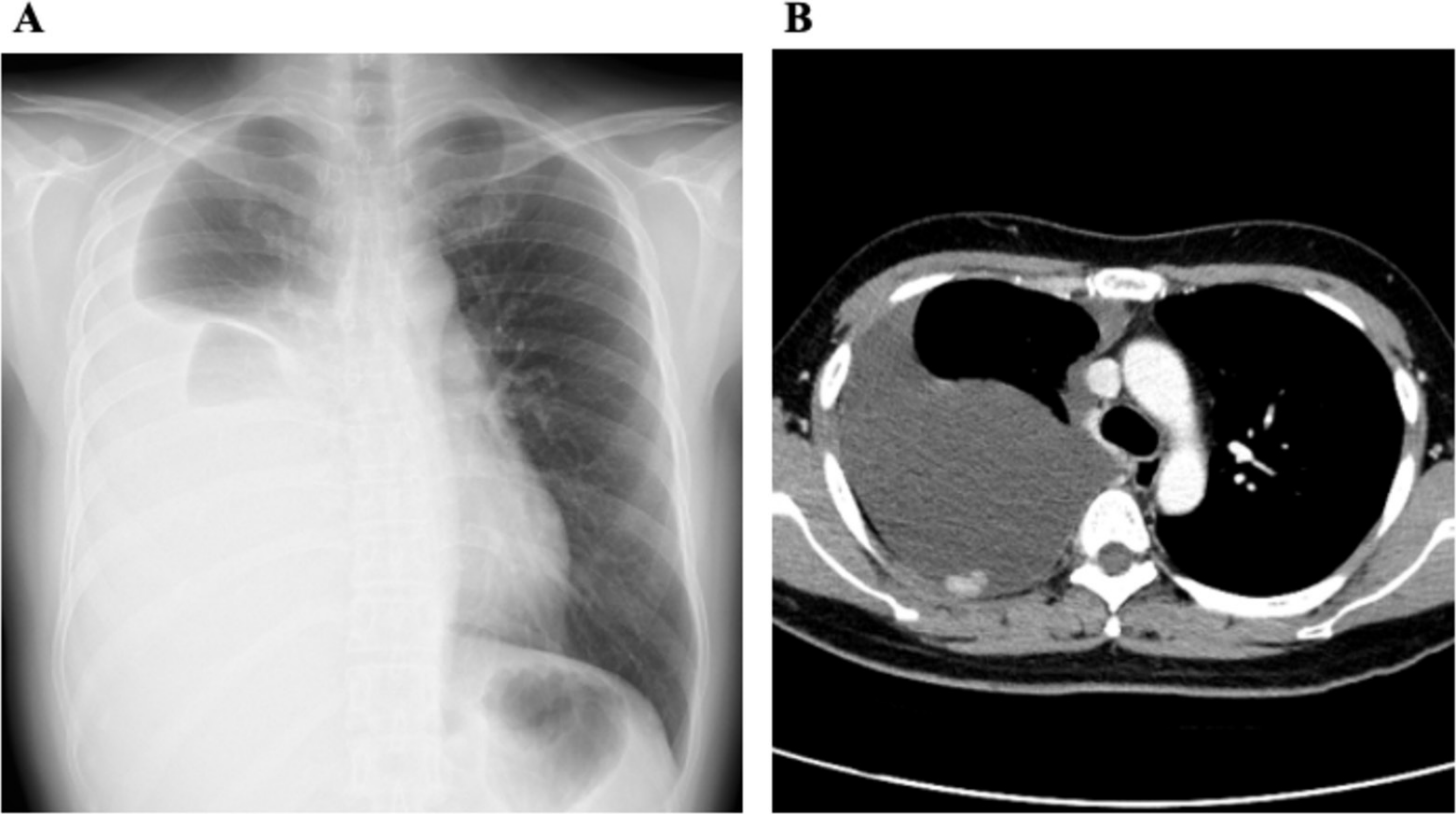
(A) Chest radiograph showing a large right‐sided pleural effusion. (B) Contrast‐enhanced chest CT demonstrating a massive right pleural effusion and multiple contrast‐enhancing nodular lesions along the pleural surface.

**FIGURE 2:**
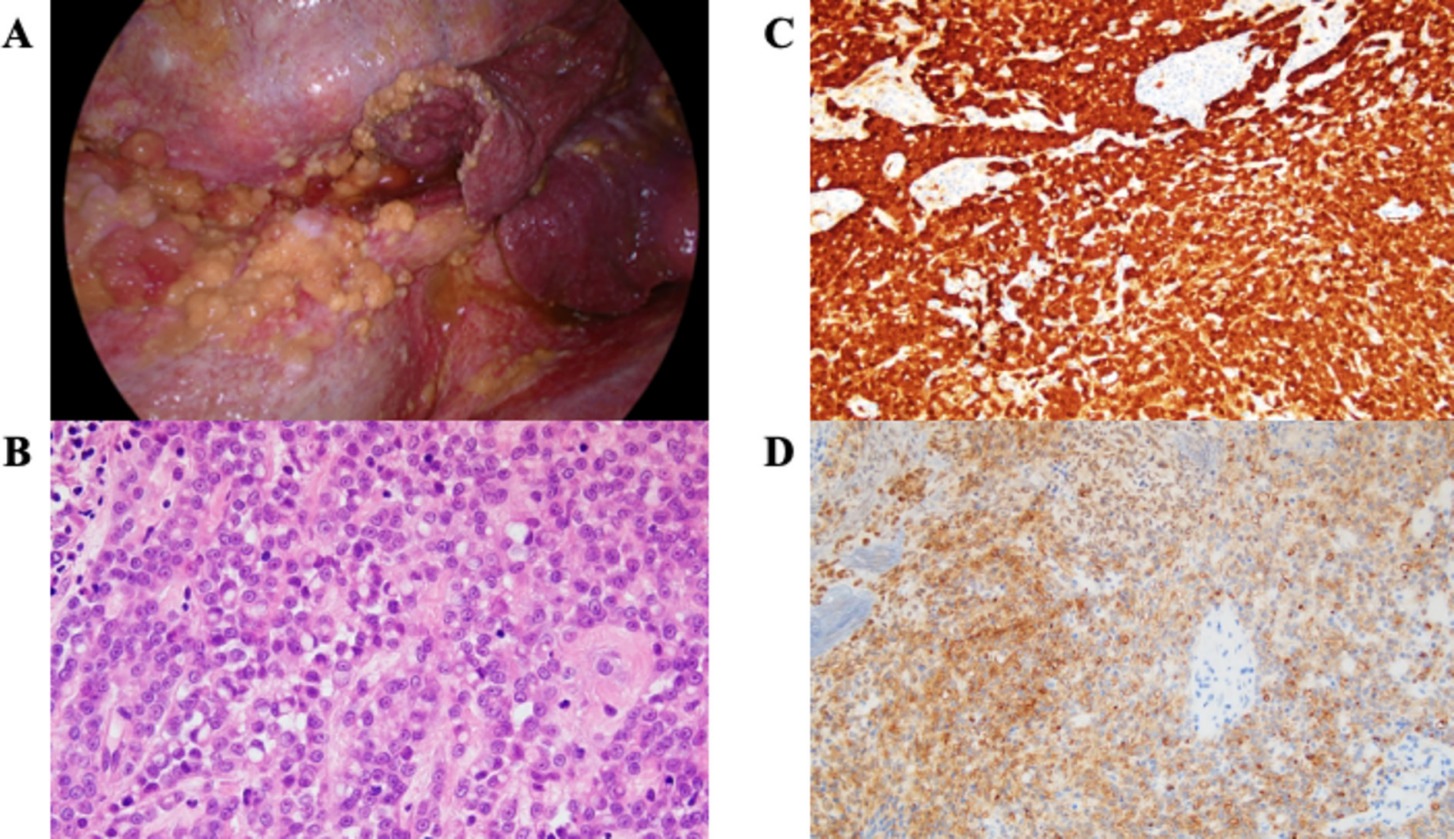
(A) Thoracoscopic view showing multiple nodular lesions on the visceral and parietal pleura. (B) Haematoxylin and eosin (HE) staining showing epithelioid tumour cells infiltrating the pleura. (C) Immunohistochemical staining showing calretinin positivity in tumour cells. (D) Immunohistochemical staining showing D2‐40 positivity in tumour cells.

A 41‐year‐old woman with a history of AML, who was treated at the age of 8 years, presented to a clinic with progressive dyspnea over the past month. A chest radiograph revealed right‐sided pleural effusion, leading to a referral to our hospital. Chest radiography and computed tomography (CT) confirmed a large right pleural effusion (Figure 1A,B). Diagnostic thoracentesis revealed an exudative pleural effusion with lymphocyte predominance, and cytology suggested the presence of tumour cells consistent with PM. A detailed interview did not confirm a history of asbestos exposure. The patient's family history included bile duct cancer in her paternal grandfather and gastric cancer in her maternal grandfather. Video‐assisted thoracoscopic surgery was performed to establish a definitive diagnosis, and multiple nodular lesions on the parietal and visceral pleura were biopsied (Figure 2A). Immunohistochemical staining of the tumour cells was positive for calretinin, D2‐40, and WT‐1 and negative for TTF‐1 and CEA, thus confirming the diagnosis of epithelioid‐type PM (Figure 2B–D). Since there was no history of exposure to asbestos, we suspected the effects of childhood leukaemia treatment and collected detailed treatment information from other hospitals where the patient had been treated, revealing that, in addition to chemotherapy, the patient had received TBI and an autologous bone marrow transplant. The patient was referred to a tertiary care hospital for multidisciplinary treatment.

## 3 Discussion

PM is most commonly associated with asbestos exposure, which remains the most well‐established etiological factor. However, a subset of PM incidences occurs in individuals without a known history of asbestos exposure. In non‐asbestos‐related cases, prior radiation therapy for other malignancies has been implicated as a potential risk factor. Several reports have described radiation‐induced PM, particularly in patients treated for Hodgkin lymphoma, non‐Hodgkin lymphoma, or breast cancer.

Previously, Chirieac et al. analysed 1618 patients with PM and identified 22 with a history of radiation therapy for lymphoma [3]. These radiation‐associated PM cases exhibited clinical characteristics different from those of asbestos‐related PM, including a significantly younger median age at diagnosis (45 years vs. 64 years, *p* < 0.001) and a longer median survival (32.5 months vs. 12.7 months). These findings suggest that radiation‐associated PM may represent a biologically distinct subtype of the disease.

Recently, BAP1 tumour predisposition syndrome (BAP1‐TPDS), a hereditary cancer syndrome caused by germline BAP1 mutations, has been recognised as another cause of mesothelioma. Typical associated tumours include mesothelioma, uveal melanoma, cutaneous melanoma, renal cell carcinoma, and basal cell carcinoma. Although cholangiocarcinoma has been reported as a less common manifestation of BAP1‐TPDS, the family history in our case was limited to bile duct cancer in the paternal grandfather and gastric cancer in the maternal grandfather, without a history of core BAP1‐associated tumours. Taken together, BAP1‐TPDS was considered less likely in this patient.

To our knowledge, only one patient with PM following treatment for leukaemia has been previously reported, and the patient underwent TBI as part of leukaemia therapy [2]. TBI is often used as a conditioning regimen for allogeneic haematopoietic stem cell transplantation (allo‐HSCT), particularly for AML. Although allo‐HSCT has significantly improved survival outcomes in haemato‐logic malignancies, long‐term survivors remain at risk of secondary malignancies, including solid tumours, such as PM [4].

Here, we report the development of PM in a 41‐year‐old long‐term leukaemia survivor with a history of childhood leukaemia and TBI. This observation highlights the importance of considering prior radiation therapy, including TBI, as a contributing factor in the development of secondary PM, especially in younger patients without a history of asbestos exposure.

Limited prognostic data are available for young patients with PM. Some studies have suggested that a younger age at diagnosis may be associated with better outcomes, potentially due to fewer comorbidities and a more favourable response to treatment [5]. However, optimal treatment strategies for this subgroup remain unclear and require further investigation. This case report emphasises the need for the long‐term follow‐up of childhood cancer survivors, especially those who have received thoracic irradiation or TBI. Although rare, PM should be considered as a potential late complication, and awareness among clinicians is essential to facilitate early diagnosis and appropriate management.

## Author Contributions

Sho Saeki contributed to the conception and drafting of this manuscript. Rin Kurosawa, Akihiro Yamada, Minori Shimamura, Takahiro Tashiro, Megumi Inaba, and Akiko Sakagami contributed to the literature review and figure preparation. Naomi Hirata, Yusuke Tomita, and Takuro Sakagami critically reviewed and edited the manuscript. Yasumiko Jodai, Shinji Iyama, Yusuke Tomita and Takuro Sakagami were responsible for patient care and clinical management. All the authors approved the final version of the manuscript and met the ICMJE criteria for authorship.

## Consent

The authors declare that written informed consent was obtained for the publication of this manuscript and accompanying images using the form provided by the Journal.

## Conflicts of Interest

The authors declare no conflicts of interest.

## Data Availability

Data sharing not applicable to this article as no datasets were generated or analysed during the current study.
